# Effect of *Allium cepa* on LAC1 gene expression and physiological activities in *Cryptococcus neoformans*

**DOI:** 10.18502/cmm.7.1.6241

**Published:** 2021-03

**Authors:** Seyed Afzal Musavinasab-Mobarakeh, Masoomeh Shams-Ghahfarokhi, Mehdi Razzaghi-Abyaneh

**Affiliations:** 1 Department of Mycology, Faculty of Medical Sciences, Tarbiat Modares University, Tehran, Iran; 2 Department of Mycology, Pasteur Institute of Iran, Tehran, Iran

**Keywords:** *Allium cepa*, Antifungal activity, *Cryptococcus neoformans*, Laccase, *LAC1* gene expression

## Abstract

**Background and Purpose:**

This study aimed to investigate the effects of *Allium cepa* ethanolic extract (EAC) on *Cryptococcus neoformans* biological activities and *LAC1* gene expression.

**Materials and Methods:**

The minimum inhibitory concentration (MIC) of EAC was determined based on the Clinical and Laboratory Standards Institute M27-A4 method at a concentration range of 125-4000 µg/ml. The EAC synergism activity was determined in combination with fluconazole (FCZ) as an antifungal azole. Laccase activity, melanin production, and cell membrane ergosterol content of *C. neoformans* were assessed at the 0.5× MIC concentration of EAC (1000 μg/ml) and FCZ (64 μg/ml) by approved methods. The expression of the *LAC1* gene was studied in the fungus exposed to 0.5× MIC concentration of EAC and FCZ using the real-time polymerase chain reaction.

**Results:**

Based on obtained results, MIC of EAC and FCZ were 2000 and 128 μg/ml, respectively. A combinatory effect was reported for FCZ and EAC by a fractional inhibitory concentration index of 0.25. The cell membrane ergosterol content was inhibited in EAC- and FCZ-treated *C. neoformans* by 58.25% and 49.85%, respectively. The laccase activity and melanin production were reduced in EAC-treated *C. neoformans* by 45.37% and 51.57%, and in FCZ-treated fungus by 54.64% and 53.68%, respectively. The expression of fungal *LAC1* at messenger RNA (mRNA) level was measured 0.46 and 0.58 folds and significantly decreased in both EAC- and FCZ-treated *C. neoformans* at the 0.5×MIC concentration, respectively (*P*<0.05).

**Conclusion:**

The findings revealed that EAC contains inhibitory compounds which interact with biological activities in *C. neoformans* and thereby, it could be considered as a potential source for the development of novel antifungal drugs.

## Introduction

Cryptococcosis caused by *Cryptococcus* spp. is considered a life-threatening fungal infection in immunocompromised patients [ 1 , 2 ]. The *Cryptococcus neoformans* is responsible for the majority of cases of cryptococcosis and possesses virulence factors, including polysaccharide capsule, melanin, and secretory enzymes production, and rapid growth at 37˚C which provide the fungus with advantages that help it reside in host cells [ 3 , 4 ]. Two enzymes, namely phenoloxidase and laccase, are involved in melanin production by the fungus which is accumulated in the fungal cell wall and protects it from oxidative stress [ 4 , 5 ]. The genes *LAC1* and *LAC2* encode two laccases, and *LAC1* is essential for melanin production [ 3 , 5 ]. 

Amphotericin B and azoles, effective drugs for clinical cryptococcosis, are reported to have considerable side effects and lead to the emergence of resistant strains [ 6 ]. The concerns about the toxicity and limited availability of these drugs have stimulated the search for natural therapeutic alternatives alone or in combination which provide a broader spectrum of action, greater potency, and reduction in the number of resistant organisms [ 7 - 11 ]. The virulence-associated enzymes are introduced as potential targets for the discovery of antifungal drugs in medicinal plants [ 12 - 15 ].

*Allium cepa* (*A. cepa*; Onion) belongs to the Liliaceae family and is known as a traditional plant with important medicinal properties. These properties have been mainly attributed to organosulfur compounds as well as a wide array of other chemically different ingredients [ 16 - 18 ]. According to the World Health Organization, this traditional plant has been widely used for the treatment of various disorders, such as bruises, colic, high blood pressure, jaundice, and even varicose veins [ 19 - 22 ]. Although the plant has been shown to have antimicrobial activity against a wide array of microorganisms, including bacteria, parasites, viruses, and fungi [ 23 - 29 ], little has been documented about its mode of antimicrobial action. 

The present study aimed to investigate the effect of ethanolic extract of *A. cepa* (EAC) on major physiological factors of *C. neoformans*, including growth, cell membrane ergosterol (CME), laccase activity, melanin production, and *LAC1* gene expression as an important fungal virulence factor. 

## Materials and Methods

### Fungal strain

This research was approved by the Ethics Committee of Tarbiat Modares University (ethics code: IR.MODARES.REC.1394.021). A clinical strain of *C. neoformans* PFCC 93-589 was obtained from the Pathogenic Fungi Culture Collection of the Pasteur Institute of Iran ( http://fa1.pasteur.ac.ir/pages.aspx?id=1152) and maintained on Sabouraud dextrose agar (SDA, Merck, Germany) for 48 h at 37°C.

### Preparation of aqueous and alcoholic extracts of Allium cepa

For the purposes of the study, 1,000 g of fresh onion white bulbs were mixed by blender and dried by a freeze-dryer (Christ, Germany). To prepare *A. cepa* extracts, 50 g of the dried powder were mixed with 250 ml of each ethanol, methanol, and sterilized distilled water, separately and sonicated at 120 Htz for one h by a sonicator (Hielscher UP400S Ultrasonic, Germany). After incubation at room temperature for 48 h on a shaker at 120 rpm, the extracts were filtered through Whatman No. 1 filter paper (Merck, Germany) to remove the debris. Moreover, ethanol and methanol extracts were dried by a rotary evaporator at 40°C [ 28 ]. The aqueous extract was concentrated to dryness using a freeze drier. The yield percentage was calculated using the following formula: 

Extract yield (%)=R (the dry weight of the extracted plant)/S (the raw plant sample)×100

### Screening of antifungal activity of Allium cepa extracts

Agar well diffusion technique was used to screen and determine the antifungal activities of *A. cepa* extracts. The final concentration of each extract was prepared in sterile distilled water at 4000 μg/ml. The fungal suspension (2.5×10^3^  colony-forming unit [CFU]/ml) was cultured on SDA medium and 100 μl of each extract that contained 400 μg dry material was inoculated in wells created on the plates and stored at 35°C for 48 h. The EAC that showed the strongest inhibitory effect against the fungal growth was selected for further steps.

### Evaluation of Allium cepa ethanolic extract antifungal activity

Antifungal activity was measured according to the guidelines of the National Committee for Clinical Laboratory Standards CLSI M27-A4 method [ 29 ]. A 100 µl cell suspension of *C. neoformans* (0.5–2.5 × 10^3^ CFU/ml) prepared in RPMI-1640 (Sigma-Aldrich, USA) plus MOPS (3-(N-morpholino) propanesulfonic acid) medium were added to each well of a 96-wells microplate. The EAC was prepared in dimethylsulfoxide (DMSO, Sigma-Aldrich) and two-fold serial dilutions were prepared in RPMI in a microplate to obtain the final concentrations of 125 to 4000 μg/ml. For fluconazole (FCZ), serial two-fold concentrations of 0.5-256 µg/ml were prepared from a stock solution of the drug (Sigma-Aldrich, USA) in methanol (Merck, Germany). Microplates were incubated at 35°C for 72 h. 

The minimum inhibitory concentrations (MIC) of EAC and FCZ were defined based on the inhibition of fungal growth in 96 well microplates by visual assay. For determining the minimum fungicidal concentration (MFC) of EAC, 50 µl of the contents of wells with no visual fungal growth was cultured on SDA plates and incubated at 37°C for 48 h. The MIC was defined as the lowest concentration of the EAC capable of interrupting any visible fungal growth. The MFC of EAC was the lowest concentration of the extract in which no fungal colony appeared on SDA plates. The MICs of FCZ were defined as the concentrations of the drug that constantly reduced the turbidity, compared to the control group.

### Effect of Allium cepa ethanolic extract on fractional inhibitory concentration index

Fractional inhibitory concentration indexes (FICI) of EAC and FCZ were determined by checkerboard titration assay at the above-mentioned concentrations [ 30 ]. Briefly, 100 µl fresh fungal cell (0.5–2.5×10^3^ CFU/ml) in RPMI-1640 was inoculated into 96-well plates containing a combination of 50 μl of FCZ and 50 μl of EAC (1:1 v/v) and incubated at 35°C for 72 h. The interactions were done using the following formula:

[(MIC of FCZ combined with EAC/MIC of FCZ alone) + [(MIC of EAC combined with FCZ/MIC of EAC alone)

The FICI results were categorized as synergy (≤0.5), additive (0.5-4.0), and antagonistic (≥4.0) [ 31 ].

### Effect of Allium cepa ethanolic extract on the ergosterol content

The ergosterol content was measured according to the instructions of Arthington-Skaggs et al. [ 32 ]. The resultant heptane extract was scanned from 200 to 300 nm using a UV-visible spectrophotometer (Shimadzu, Japan) and the absorbance of the samples was read at two wavelengths of 281.5 nm (A_281.5_) and 230 nm (A_230_). The ergosterol content was calculated as a percentage of the wet weight of the cell using the following formula:

Ergosterol (%)= [(A_281.5_/290) × F/fungal pellet weight] – [(A_230_/518) × F/fungal pellet weight]

F is the dilution factor, 290 and 518 are the E-values for crystalline ergosterol and 24 (28) dehydroergosterol, respectively. 

### Effect of Allium cepa ethanolic extract on melanin production

Melanin production was assessed in *C. neoformans* (2.5×10^6^ cells/ml) exposed to EAC (1000 μg/ml) and FCZ (64 μg/ml) in 250 ml flasks containing yeast extract-peptone-dextrose (YPD) (1% w/v yeast extract, 2% w/v peptone, 2% w/v dextrose, and 1 mM L-dopa) broth medium according to the instructions given by Ikeda et al. (2003). The final processed samples were kept in hydrochloric acid (6 M) at 100°C for 30 min, and relative absorbance units from 200 to 900 nm were measured as indicators of melanin existence [ 33 ].

### Effect of Allium cepa ethanolic extract on laccase activity of Cryptococcus neoformans

Laccase activity was measured in *C. neoformans* (2.5×10^6^ cells/ml) exposed to EAC (1000 μg/ml) and FCZ (64 μg/ml) in 250 ml flasks containing YPD broth according to the instructions given by Albino et al. [ 34 ]. The enzyme unit was defined as the amount of enzyme which oxidized one µmol of substrate per min in the assay conditions. 

### Effect of Allium cepa ethanolic extract on the LAC1 gene expression

Expression of the *LAC1* gene, as a mediator for the biosynthesis of laccase, was studied by a quantitative polymerase chain reaction (PCR) using specific primers of *C. neoformans* exposed to 0.5× MICs of EAC (1000 μg/ml) and FCZ (64 μg/ml). Total RNA was extracted by the RNX-Plus Kit (Sinacolon, Iran). Moreover, cDNA synthesis was carried according to the directions given by Jahanshiri et al. [ 35 ] using the primers *LAC1*; F: 5ʹ-AGAAGGGAAGGAAGGTGATG-3ʹ, Reverse: 5ʹ-TATACCTCACAACCGCCAA-3ʹ, and ACT1 (Forward: 5ʹ-TGCCTCTGGTCGTACCACTG-3ʹ and Reverse: 5ʹ-GCGAAACCTTCGTAGATGGG-3ʹ) [ 36 ]. 

It should be mentioned that amplification products were analyzed by agarose (1%) gel electrophoresis. To calibrate the real-time quantitative reverse transcription PCR system, a standard curve from serial dilutions (10^−1^ to 10^−4^) of the cDNA template of *C. neoformans* was used. Real-time PCR was carried out using SYBR green master mix (Applied Biosystems, USA) using the *β-actin* as a reference gene. The PCR was performed with an initial incubation at 95°C for 10 min, followed by 40 cycles of denaturation (95°C for 15 sec), annealing (60°C for 1 min) and extension (72°C for 15 sec) and a final extension step at 72°C for 1 min by the ABI PRISM 7500 thermal cycler (Applied Biosystems, USA). To determine the mRNA level of *LAC1* expression, the differences between the threshold cycle (CT) of samples and calibrator [ΔCT=CT (target) - CT (reference)] were calculated using the following formula: (2^−ΔΔCT^) and analyzed by REST© software (version 2.0.13). 

### Statistical analysis

All data were analyzed in GraphPad Prism software (version 6.0, Sandiego, CA). A p-value of less than 0.05 was considered statistically significant in all the analyses.

## Results

### Antifungal activity of Allium cepa

Screening results on agar well diffusion assay showed that EAC (15 mm) was more effective than the aqueous (2 mm) and methanolic (10 mm) extracts which were used for all the next steps in this study. The MIC and MFC values of EAC were determined in comparison with FCZ against the *C. neoformans* (Table 1). Results indicated that MIC and MFC of EAC against *C. neoformans* were 2000 and 4000 μg/ml, respectively. For FCZ, these values were reported at 128 and 256 μg/ml, respectively. The FICIs for EAC combined with FCZ were calculated as well. Results of the checkerboard microtiter assay indicated significant synergistic interaction between EAC and FCZ against *C. neoformans* (Table 1). The synergistic interaction between EAC and FCZ (0.5>FICI=0.25) against *C. neoformans* was observed after 72 h of incubation at 35°C.

**Table1:** Minimum inhibitory concentration (MIC), minimum fungicidal concentration, and fractional inhibitory concentration index (FICI) of *Allium cepa* ethanolic extract (EAC) and fluconazole (FCZ) against *Cryptococcus neoformans*

Antifungal	MIC (μg/ml)				MIC without combination	MIC with combination	FICI
	Range	MIC_50_	MIC_90_	MFC			
EAC	125-4000	1000	2000	4000	2000	500	0.25
FCZ	0.5-256	64	128	256	128	0.5	

### Effect of Allium cepa ethanolic extract on cell membrane ergosterol content

The CME contents of *C. neoformans* in EAC- and FCZ-treated cultures were measured at 0.42 and 0.50 µg/g, respectively (Table 2). The CME was inhibited by 58.25% and 49.85% in EAC- and FCZ-treated cultures, respectively. The results showed that FCZ and EAC were able to efficiently reduce ergosterol content in treated *C. neoformans*. A significant difference was observed between the treated and control groups (*P*<0.001).

**Table2:** Effects of *Allium cepa* ethanolic extract (EAC) and fluconazole (FCZ) on fungal cell weight and ergosterol content in *Cryptococcus neoformans*

Groups	Fungal dry weight (mg) Mean±SD	Fungal growth inhibition (%)	Ergosterol content (µg/g) Mean±SD	Inhibition of ergosterol (%)
EAC-treated	0.40±0.01	61.90	0.422±0.067	58.25
FCZ-treated	0.62±0.05	40.95	0.507±0.012	49.85
Non-treated control	1.05±0.02	0.0	1.011±0.010	0.0

### Effect of Allium cepa ethanolic extract on melanin production and laccase activity

**Figure 1:**
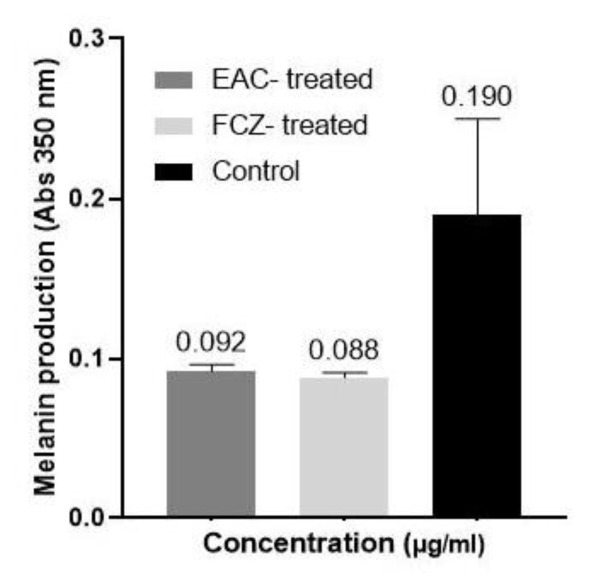
Melanin production in *Cryptococcus neoformans* treated with ethanolic extract of *Allium cepa* (EAC) and fluconazole (FCZ) (0.5× MIC; µg/ml)

**Figure 2:**
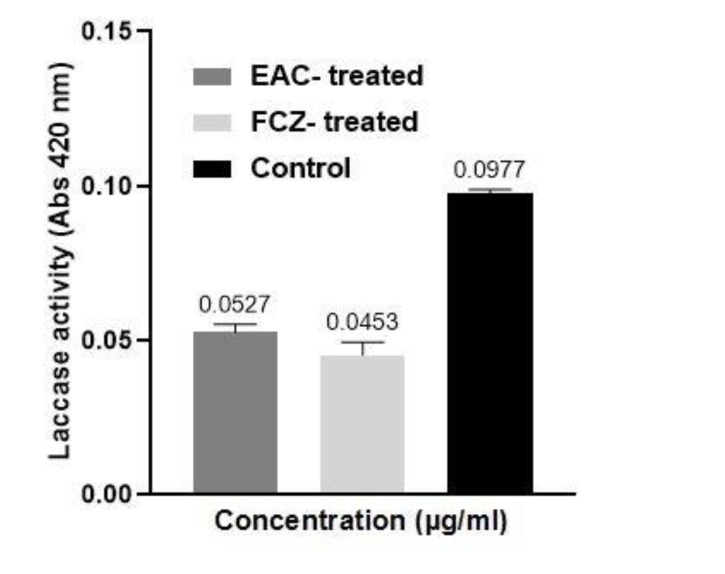
Laccase activity in *Cryptococcus neoformans* treated with ethanolic extract of *Allium cepa* (EAC) and fluconazole (FCZ) (0.5× MIC; µg/ml)

Melanin production decreased in *C. neoformans* treated with 0.5× MICs of both EAC (0.092) and FCZ (0.088) as summarized in Table 3. The melanin production was significantly inhibited by 51.57% and 53.68% in EAC- and FCZ-treated cultures, compared to the control group (*P*<0.05) (Figure 1). The laccase activity was measured at 0.053 and 0.044 in *C. neoformans* exposed to EAC and FCZ, respectively (Table 3). Furthermore, the enzyme activity was inhibited by 45.37% in EAC-treated and 54.64% in FCZ-treated cultures. The results showed that both EAC and FCZ were able to significantly reduce laccase activity in *C. neoformans*, compared to the control group (*P*<0.05) (Figure 2).

**Table3:** Effects of *Allium cepa* ethanolic extract (EAC) and fluconazole (FCZ) on laccase activity and melanin production in *Cryptococcus neoformans*

Groups	Laccase activity (µmol/min) Mean±SD	Inhibition of laccase activity (%)	Melanin production (Abs 350 nm) Mean±SD	Inhibition of melanin production (%)
EAC-treated	0.053±0.02	45.37	0.092±0.004	51.57
FCZ-treated	0.044 ±0.03	54.64	0.088±0.003	53.68
Non-treated control	0.097±0.01	0.0	0.19±0.060	0.0

### Effect of Allium cepa ethanolic extract on LAC1 gene expression

**Figure 3:**
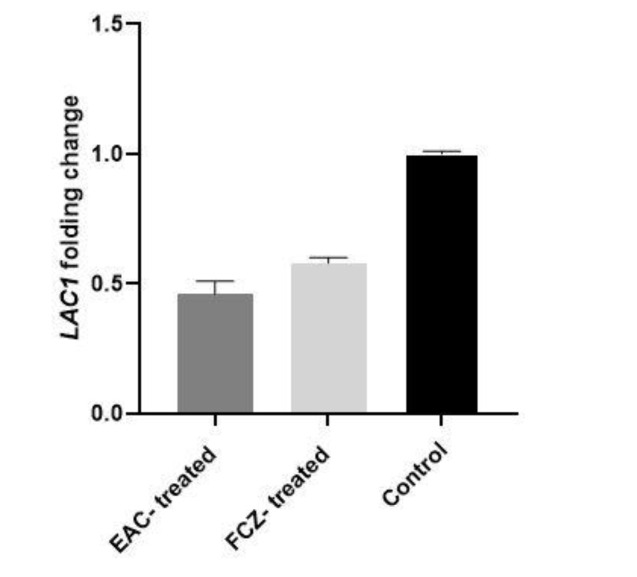
Comparison of the expression of *LAC1* at messenger RNA level between the control group and the group of *Cryptococcus neoformans* treated with ethanolic extract of *Allium cepa* (EAC) and fluconazole (FCZ) (*P*<0.05).

The relative quantification of the *LAC1* expression ratio normalized to *β-actin* indicated that there were significant differences between the gene expressions of EAC- and FCZ-treated fungal cells and the untreated control group. Real-time PCR demonstrated that the expression levels of *LAC1* in all the treated samples with 0.5× MIC concentration of EAC (1,000 μg/ml) and FCZ (64 μg/ml) were 0.43 and 0.53 folds lower than the control samples, respectively (Figure 3). The mRNA transcripts level of the *LAC1* gene underwent a significant decrease and down-regulation in comparison with non-treated control samples (*P*<0.05). 

## Discussion

To clarify the exact mode of antifungal action of *A. cepa* against *C. neoformans*, we evaluated the potential targets of EAC *in vitro*. For this purpose, the fungal plasma membrane ergosterol synthesis, laccase activity, melanin production, and *LAC1* gene expression were studied in *C. neoformans* exposed to *A. cepa*. 

Several studies have confirmed the antifungal activity of white onion against various pathogenic fungi. Lanzotti et al. found the three ceposides extracted from white onion strongly inhibited the growth of some filamentous fungi at 200 ppm concentration [ 25 ]. Kocić‐Tanackov et al. demonstrated the fungicidal effect of onion essential oil at a concentration of 28.0 µl/100 ml on the growth of some spp. that belonged to the genera *Aspergillus*, *Penicillium*, and Fusarium [ 26 ]. Balamanikandan et al. revealed that the onion-based synthesized nanoparticles had significant antifungal activity against various *Aspergillus* spp. [ 27 ]. Ikegbunam et al. reported the antifungal activities of the aqueous and alcoholic extracts of *A. cepa* against *C. albicans*, *Aspergillus niger*, and *Trichophyton rubrum* [ 28 ]. Based on the results of another study, the organic ethanol extracts of onion (*A. cepa*) (MFC: 275 mg/ml) inhibited the growth of *Aspergillus flavus*, *A. niger*, and *Cladosporium herbarum* [ 37 ]. 

Shams-Ghahfarokhi et al. [ 38 ] also found ultrastructural cell damage in *T. rubrum* and *Trichophyton mentagrophytes* after exposure to fresh *A. cepa* aqueous extract in a concentration of 200 mg/ml. They indicated different changes in the fungal cytoplasmic membranes and other membranous structures of organelles, such as nuclei and mitochondria, cell wall, complete or partial destruction of organelles, degeneration of the cytoplasm, and formation of electron-dense material in hyphae cells which led to cell death. In another study, the zones of inhibitions are shown by gel of red onion at different concentrations (5, 7.5, and 12.5%) against *T. rubrum* [ 39 ]. The gel with a concentration of 12.5% showed significant antifungal activity against *T. rubrum* (10–20 mm) (*P*<0.05). 

In this study, the screening results showed that EAC was more effective than the aqueous and methanolic extracts against *C. neoformans*. The EAC inhibited the fungal growth by 50% at 1,000 μg/ml while the fungal growth was completely inhibited at 4,000 μg/ml. The EAC at a concentration of 125 μg/ml increased the sensitivity of *C. neoformans* to FCZ (MIC: 0.5 μg/ml). The FICI values of EAC in combination with FCZ was reported at 0.25 which shows a combinatory effect for these compound. Moreover, it was found that EAC in the concentration of 1,000 µg/ml decreased the cell membrane ergosterol content to 0.42 µg/g fungal dry weight in the treated *C. neoformans*. The EAC probably increases the susceptibility of *C. neoformans* to FCZ by changing the cell membrane ergosterol content. Reduced ergosterol content interferes with the permeability of fungal cells and causes the release of various important components from inside of the fungal cell.

Sulfhydryl organic compounds have been reported to be laccase inhibitors. Nickavar and Yousefian found that ethanol extracts of *Allium spp*. were able to inhibit α-amylase activity with IC_50_ values around 15 mg/ml [ 40 ]. In this study, the laccase activity and consequently, melanin production was potently reduced in *C. neoformans* exposed to both EAC and FCZ. This inhibitory effect may be attributed to the direct effect of EAC on laccase activity or may be the consequence of the release of important fungal cell components essential for the biosynthesis of laccase after changing fungal cell membrane permeability by EAC.

To our knowledge, this is the first report on the effect of EAC on *LAC1* gene expression in *C. neoformans*. The *LAC1* gene has been shown to be important as one of the crucial steps of melanin biosynthesis in *C. neoformans*. Results of this study showed that *LAC1* expression underwent a significant reduction in *C. neoformans* exposed to EAC (1000 μg/ml) and FCZ (64 μg/ml) by 0.46 and 0.58 folds, respectively. 

## Conclusion

In conclusion, the findings of this study indicated the effectiveness of EAC against *C. neoformans* through affecting the cell membrane ergosterol content, laccase activity, melanin production, and *LAC1* gene expression. The EAC could be considered a potential source for novel antifungal drugs which may be effective in the treatment of invasive cryptococcosis. Since biological activities of EAC against *C. neoformans* may be attributed to flavonoids and organosulphur compounds as two major classes of phytochemicals of *A. cepa*, further studies are recommended to isolate and characterize antifungal components of EAC.

## Authors’ contribution

M. S. G. conceived and designed the study. S. A. M. carried out the experiments. M. S. G., S. A. M., and M. R. A. carried out the data analysis, wrote and revised the manuscript. All authors read and approved the manuscript. 

## Financial disclosure

The authors have no relevant financial disclosures relevant to this article.
